# A Modelling Approach to Estimate the Impact of Sodium Reduction in Soups on Cardiovascular Health in the Netherlands

**DOI:** 10.3390/nu7095375

**Published:** 2015-09-18

**Authors:** Maaike J. Bruins, Mariska Dötsch-Klerk, Joep Matthee, Mary Kearney, Kathelijn van Elk, Peter Weber, Manfred Eggersdorfer

**Affiliations:** 1 DSM Biotechnology Center, Alexander Fleminglaan 1, Delft 2613AX, The Netherlands; 2 Unilever R&D Vlaardingen, Olivier van Noortlaan 120, Vlaardingen 3133 AT, The Netherlands; E-Mails: Mariska.Dotsch@unilever.com (M.D.-K.); Joep.Matthee@unilever.com (J.M.); Mary.Kearney@unilever.com (M.K.); Kathelijn-van.Elk@unilever.com (K.E.); 3 DSM Nutritional Products, Wurmisweg 576, Kaiseraugst CH-4303, Switzerland; E-Mails: peter.weber@dsm.com (P.W.); manfred.eggersdorfer@dsm.com (M.E.)

**Keywords:** blood pressure, cardiovascular disease, food reformulation, salt, sodium, public health, health burden, disability-adjusted life years

## Abstract

Hypertension is a major modifiable risk factor for cardiovascular disease and mortality, which could be lowered by reducing dietary sodium. The potential health impact of a product reformulation in the Netherlands was modelled, selecting packaged soups containing on average 25% less sodium as an example of an achievable product reformulation when implemented gradually. First, the blood pressure lowering resulting from sodium intake reduction was modelled. Second, the predicted blood pressure lowering was translated into potentially preventable incidence and mortality cases from stroke, acute myocardial infarction (AMI), angina pectoris, and heart failure (HF) implementing one year salt reduction. Finally, the potentially preventable subsequent lifetime Disability-Adjusted Life Years (DALYs) were calculated. The sodium reduction in soups might potentially reduce the incidence and mortality of stroke by approximately 0.5%, AMI and angina by 0.3%, and HF by 0.2%. The related burden of disease could be reduced by approximately 800 lifetime DALYs. This modelling approach can be used to provide insight into the potential public health impact of sodium reduction in specific food products. The data demonstrate that an achievable food product reformulation to reduce sodium can potentially benefit public health, albeit modest. When implemented across multiple product categories and countries, a significant health impact could be achieved.

## 1. Introduction

High blood pressure is the 5th leading risk factor of the global burden of disease in terms of Disability-Adjusted Life Years (DALYs) [1]. Blood pressure is known to be a major modifiable risk factor for cardiovascular disease, including stroke and Ischemic Heart Disease (IHD) [2,3]. Randomized controlled trials have demonstrated that reducing sodium intake lowers blood pressure in a linear way [4,5]. Moreover, evidence from prospective cohort studies indicates a relationship between higher levels of sodium intake and increased risk of cardiovascular disease [5]. Therefore, it is assumed that reductions in sodium ultimately result in lower risk of cardiovascular disease.

The estimated mean global daily intake of 4 g sodium (10 g salt) [6] largely exceeds the current World Health Organization (WHO) daily recommendation of less than 2 g sodium (5 g salt) [7]. Reducing sodium intakes is therefore a recognized public health priority of health authorities and non-governmental organizations. In European and North American countries, generally over 75% of sodium intake comes from food products (mostly meat, bread and other cereal products, but also cheese and soups). In contrast, in Asian countries discretionary salt contributes most to sodium intake [8].

Reducing sodium in food products represents a technical challenge as salt is added to food to enhance taste, increase product shelf life and improve texture [9]. Consumers may not accept significantly lower salt levels in food products as was shown for soups [10]. The most promising strategy is to adapt consumer’s preferences for saltiness by reducing sodium in products in small steps [10]. However, this approach needs governmental and non-governmental organizations, health care professionals, and the food industry to collaborate in order to be effective. Therefore, the food industry is also exploring other solutions to limit sodium content in food products in order to provide healthier products that help people improve their health. Examples include the use of mineral salts with less or no sodium, and natural taste enhancers such as yeast extracts, aromas, and spices. Currently applied approaches are resulting in sodium reductions between 20%–30% depending on the application [11]. A joint effort to reformulate food products to reduce the levels of salt will help consumers achieving a healthier diet.

In this paper, to explore the potential health impact of a potentially achievable sodium reduction in a specific food, a modelling approach was applied to estimate the preventable DALYs related to cardiovascular disease. In this modelling study, sodium reduction in packaged soups in the Netherlands was selected as a widely consumed food category in which successful sodium reduction can be achieved if carried out gradually and industry-wide.

## 2. Modelling Methods

Data from the Dutch National Food Consumption Survey 2007–2010 [12] and the Dutch Food Composition Database 2011 [13] were used to collect data on average soup consumption, and sodium content of soup, respectively. An average 25% sodium reduction in soup, was considered achievable over time when executed gradually [14]. The effect of 25% sodium reduction in soup in 2011 on cardiovascular-related morbidity and mortality in that year and subsequent lifetime effects was simulated for the adult Dutch population aged ≥20 years with a size of 12.7 million people (49% men, 51% women).

As a first step, the dose-response relationship between changes in sodium intake and changes in Systolic Blood Pressure (SBP) was derived from a recent meta-analysis by He *et al*. [15] showing that per 4.4 g/day reduction in salt intake (as measured by urinary sodium excretion), SBP decreases on average by 4.18 mmHg. The reductions in incidence of stroke, AMI and angina pectoris events in relation to SBP lowering were derived from Relative Risk Reductions reported per 10-years age group in a meta-analysis by Law *et al.* [2]. Systolic Blood Pressure-related reductions in mortality from stroke, AMI and angina were derived from a meta-analysis by Lewington *et al*. [3]. The reduction in SBP related incidence and mortality of Heart Failure (HF) was derived from Relative Risk Reductions reported in different studies [16,17,18,19,20,21]. For the different age groups, a log-linear relation between SBP lowering and reduction of disease mortality and incidence was assumed [3].

The DALY can be used to assess health impact; the DALY combines mortality and morbidity into a single, quantitative metric. The health burden is the sum of years of life lost due to premature mortality (YLL) and morbidity (years lived with disability; YLD). The WHO method [22] and calculation template [23] were used to calculate DALYs due to cardiovascular disease-related morbidity and mortality. The potential DALYs prevented when reducing sodium in soup were calculated as the difference in DALYs between the scenario without and with salt reduction.

Global disease disability weights and data on the population size and life expectancies, cardiovascular disease-specific incidence cases, mortality cases, and durations as reported for the Netherlands in 2011 served as inputs for the model. These data were extracted from the Statistics Netherlands (CBS) [24] and Volksgezondheid en Zorg [25] databases, all per gender and 10-year age group ≥20 years. Incidence data on cardiovascular morbidity and mortality were available for stroke (ICD-10-code I60-I69), HF (ICD-10-code I50), AMI (ICD-10-code I21-I22) and angina (ICD-10-code I20). Incidents of AMI and angina were based on reported incidence trends for AMI and angina in 2011 [25]. Stroke was assumed reversible and disability to last six months in 65% of patients, and leading to permanent moderate to severe disability in 35% of patients [26]. Netherlands-specific data were collected on time lived with an acute disability (hospital stay) for stroke [27] and AMI [28], and time lived with a permanent disability for stroke, angina, and HF were derived by dividing prevalence rates by incidence rates [25]. Frequency-weighted average disability weights for the hyperacute and acute phase of stroke [29], the hyperacute and acute phase of AMI [29], and chronic disability for angina [30] and HF [30] were based on results of Global Burden of Disease (GBD) 2010, and for disabled stroke survivors, based on GBD 2004 results [29]. Discount rate and age weight were not applied as these were recently dropped following informal consultations in 2012 by WHO [31].

## 3. Results

The median daily soup consumption of the Dutch population ≥20 years was 52.4 g with comparable consumption among age groups and genders. Given that soups in 2011 in the Netherlands contained on average 340 mg/100 g sodium, this would reflect a median daily intake of 180 mg sodium (0.46 g salt) from soup. An average of 25% sodium reduction in soups would reduce median population sodium intake by 45 mg/day (*i.e.*, salt by 0.11 g/day). Based on the dose-response relationship reported between salt intake and SBP, a 45 mg/day lower sodium intake could reduce the average SBP by 0.11 mmHg.

Based on reported Relative Risk Reductions, a reduction in SBP of 0.11 mm Hg could reduce the stroke-related incidence by (age-weighted average) 0.49%, AMI and angina incidence by 0.34%, and HF incidence by 0.24%. A 0.11 mmHg reduction was estimated to reduce on average the stroke-related mortality by 0.46%, AMI and angina mortality by 0.34%, and HF mortality by 0.24%. The related number of potential cases averted is shown in Table 1.

**Table 1:** Estimate of potentially avertable mortality and morbidity incidence cases in the 2011 Dutch population cohort aged ≥20 years, if reducing sodium in soups by 25%.

	Males	Females	Total
Total mortality	24	23	47
Stroke incidence	49	46	95
AMI incidence	56	29	85
Angina incidence	41	29	70
HF incidence	17	20	37

AMI: acute myocardial infarction; HF: heart failure.

The number of years of life lost due to premature mortality and years lived with the disease, and the total number of DALYs in the Dutch population that could potentially be averted by a 45 mg/day sodium reduction in soups are presented in Table 2. Mainly preventable stroke and angina with long-term consequences contributed to the preventable YLD whereas acute stroke and AMI contributed little considering their short-term nature (data not shown).

**Table 2:** Estimate of potentially avertable number of years of life lost (YLL), years lived with disability (YLD) and disability-adjusted life years (DALYs) (at 0% discount rate, 0% age weight) in the 2011 Dutch population cohort aged ≥20 years, if reducing sodium in soups by 25%.

	Males	Females	Total
YLL	341	257	598
YLD	123	98	221
DALYs	464	355	819

YLL: Years of Life Lost; YLD: Years Lived with Disability; DALY: Disability-Adjusted Life Years.

## 4. Discussion

This modelling study indicates that an average salt reduction in soups of 25% in The Netherlands in one year could potentially save approximately 800 lifetime DALYs related to cardiovascular disease.

Widespread recommendations to reduce salt intake with the purpose of promoting public health have led to specific global and national targets for reducing salt in manufactured foods [32]. In order to achieve these targets, small and large commitment from food industries to collectively reformulate foods is of key importance. Today, many food industries are engaged in sodium reduction, but, in many countries, salt intake is still exceeding the recommendations [32]. More commitments and partnerships are required to get intakes closer to the recommendations. Estimating the potential impact of specific initiatives can help encourage more stakeholders for greater engagement in salt reduction.

Salt reduction in soups in The Netherlands was selected as a practical example of a product group and country where data are available and sodium reduction is genuinely happening. This allows for an assessment of what impact a realistic sodium reduction in a specific food group can have on public health. An average reduction of 25% in soups is considered potentially feasible on the longer term (stressing that this concerns an *average* reduction of 25% as not all soups have the same sodium level, so cannot be reduced with the same amount) [14]. However, such a reduction is only achievable when executed in small steps as consumers will not accept too drastic changes in taste or may even add back salt themselves [10].

In this modelling study, demographics and most recent cardiovascular morbidity and mortality data from The Netherlands were used as model inputs to estimate the gain in health from salt reduction in soup. Although salt lowering might also reduce the risk related to stomach cancer [33] and chronic kidney disease [34], the data available are not sufficient to properly estimate the potential impact of salt reduction on these health outcomes.

In the current analysis, a linear relationship between salt restriction and SBP lowering was assumed as based on a meta-analysis of intervention trials in which sodium reduction was in the middle range (0.9–2.7 g/day) [15]. However, SBP lowering may not be fully linear across the range of reduced sodium intakes, smaller effects being achieved with smaller reductions, and larger effects achieved with larger reductions [35]. A premise of the current analysis is that sodium reduction from soups (45 mg/day) is part of a broader initiative of medium sodium reduction to reach the threshold for achieving a meaningful SBP reduction.

As this is a modelling approach which has its limitations, some care should be taken interpreting the findings. For instance, the estimated risk reductions were derived from relative risk reductions reported in multiple international studies, and therefore not specific for the Netherlands. In addition, relative risk reductions for cardiovascular incidence and mortality associated with SBP lowering were modelled for age groups for which no relative risk reduction was reported. Stroke is a complex syndrome including multiple types and subtypes with varying causes and outcomes, but we tried to address this as much as possible by modelling stroke with acute, medium and long-term entities.

It should also be noted that the estimate of burden of disease depends on the selected method. We selected the WHO calculation template that provides rough DALY estimates and is restricted to predicting prospective morbidity and mortality in a one-year population cohort. More sophisticated (dynamic or stochastic) Monte Carlo-based disease progression mathematical models, exist that simulate long-term effects of interventions by calculating cumulative incidence and mortality, changes in life expectancy, transition between disease states, and provide uncertainty ranges in the final DALY result.

Previous modelling studies have also shown a positive public health impact of salt reduction strategies [36,37,38,39,40]. However, as the studies differ in scenarios, inputs and methodology, direct comparisons are difficult to make. Hendriksen *et al*. [36] also simulated the health impact of salt-reduction on the adult Dutch population aged ≥20 years The authors used similar output variables (incidence and mortality of stroke, AMI, and HF), but used salt reduction scenario’s related to larger reductions in salt intake (2.3 to 3.0 g/day). In addition, the impact of salt reduction was simulated over a 20-years period using a more sophisticated model [36]. However, when similar reductions in salt intake were assumed in our model, comparable estimations of potential incidence cases averted in the first year were obtained, supporting the validity of our results. Health impact modelling in the domain of public health nutrition is still in its infancy, and there is strong need for further alignment and accessibility of practical and valid models to enable robust impact assessment of public health strategies, including product reformulation.

## 5. Conclusions

This is the first study that has estimated the potential public health impact of a substantial salt reduction in a single food category. Since considerable acute and long-term health care costs and productivity losses are involved with HF, stroke [41] and AMI [42], it is likely that reducing respective incident cases can significantly reduce related health care costs and productivity losses. This case study shows that a substantial and achievable salt reduction in soups in the Netherlands as part of an overall salt reduction strategy, could potentially have an impact, albeit small, on public health. Provided a cohesive industry-wide total diet approach is adopted, implying that sodium reduction is executed in more product groups and countries, small changes can add up to make a big difference.

## Supplementary Materials

Supplementary Materials are provided on the Modelling approach, and inputs and outputs (Table S1–Table S24, Figure S1–S3). Tables S1–S3 show the demographics data that were collected for The Netherlands in 2011. Table S4 shows the case fatality rates for the Netherlands in 2001 and Table S5 the estimated percentage of patients suffering from a disability.
