# Supplementary material for: A Modelling Approach to Estimate the Impact of Sodium Reduction in Soups on Cardiovascular Health in the Netherlands
Source: Nutrients. 2015 Sep 18;7(9):8010–9. doi: 10.3390/nu7095375 (PMC4586570; doi:10.3390/nu7095375)
Supplement: Supplementary file 1 [file nutrients-07-05375-s001.docx]

**Supplementary Materials**

# 1. General Approach Followed

The WHO tool [1] to calculate burden of disease has been used using principles of DALY calculations as described by the WHO [2].

DALY = YLD + YLL

YLL = Years of Life Lost due to premature mortality

YLD = Years Lived with Disability

YLL = N × L

*where:*

N = number of deaths (per year)

L = standard life expectancy at age of death (in years)

YLD = I × DW × L

*where:*

I = number of incident cases (per year)

DW = disability weight

L = average duration of disability (years)

| DALY *usual sodium* | DALY *lower sodium* | ∆ DALY |
| --- | --- | --- |
| DALY = YLD + YLL | DALY = YLD + YLL |  |
| YLD = I × DW × L | YLD = I × RR *morb* × DW × L |  |
| YLL = N × L | YLL = N × RR *mort* × L |  |

*where:*

RR *morb* = Relative Risk morbidity with lower sodium.

RR *mort* = Relative Risk mortality with lower sodium

**2. Sodium Reduction of Soups**

- - Based on non-consecutive 24 h dietary recall data of a Dutch National Food Consumption Survey performed between 2007–2010, Dutch adults aged 19 to 69 years consumed soup (including home-made soup) for 19.5% of the year equaling 71 days [3].
  - On those 71 days, the median soup consumption was 268.3 g/day equaling 52.4 g/day.
  - The average sodium content of soup is 0.0034 g/g soup [4] disregarding that people add additional salt in their soup.
  - Sodium intake via soup is: 0.0034 g sodium/g soup × 52.4 g soup/day = 0.18 g sodium/day (0.46 g salt/day).
  - A 25% reduction in sodium content of soup would result in 45 mg/day less sodium intake (0.11 g/day less salt).

# 3. Demographics

Tables S1–S3 show the demographics data that was collected for the Netherlands in 2011 for ages ≥ 20 years, men and women [5]. Data were grouped by 10-year age groups for ages ≥ 20 years.

**Table S1.** Population 1 January 2011 by age and sex.

| **Age (Year)** | **Total** | **Men** | **Women** |
| --- | --- | --- | --- |
| 20–29 | 2,036,267 | 1,026,784 | 1,009,483 |
| 30–39 | 2,126,332 | 1,063.612 | 1,062,720 |
| 40–49 | 2,594,217 | 1,308,966 | 1,285,251 |
| 50–59 | 2,286,566 | 1.147,992 | 1,138,574 |
| 60–69 | 1,894,212 | 944,171 | 950,041 |
| 70–79 | 1,136,839 | 521,650 | 615,189 |
| 80+ | 667,547 | 228,313 | 439,234 |

**Table S2.** Deaths 1 January 2011 by age and sex.

| **Age (Year)** | **Total** | **Men** | **Women** |
| --- | --- | --- | --- |
| 20–29 | 683 | 460 | 223 |
| 30–39 | 1199 | 706 | 493 |
| 40–49 | 3804 | 2073 | 1731 |
| 50–59 | 9391 | 5281 | 4110 |
| 60–69 | 18,773 | 11,214 | 7559 |
| 70–79 | 27,898 | 16,146 | 11,752 |
| 80+ | 69,947 | 26,992 | 42,955 |

**Table S3.** Life expectancy 1 January 2011 by age and sex.

| **Age (Year)** | **Total** | **Men** | **Women** |
| --- | --- | --- | --- |
| 20–29 | 57 | 56 | 59 |
| 30–39 | 48 | 46 | 49 |
| 40–49 | 38 | 36 | 40 |
| 50–59 | 29 | 27 | 30 |
| 60–69 | 20 | 19 | 22 |
| 70–79 | 13 | 11 | 14 |
| 80+ | 7 | 7 | 8 |

**4. Case Fatality, and Percent of Patients with a Disability**

Table S4 shows the case fatality rates for the Netherlands in 2001 of stroke, Acute Myocardial Infarction (AMI), angina pectoris, and heart failure (HF) as reported by Organisation for Economic Cooperation and Development (OECD) [6]. Table S5 lists the estimated percentage of patients suffering from a disability based on data from the Global Burden of Disease (GBD) study or the Netherlands data [7].

**Table S4.** Case fatality rates in 2011 reported by OECS [6].

| **Diagnosis** | **Case Fatality** |
| --- | --- |
| Stroke  Acute Myocardial Infarction (AMI) | 10.3%  9.8% |
| Angina | 0% |
| Heart Failure (HF) | 0% |

**Table S5.** Percent of patients with a disability.

| **Diagnosis** | **% Disabled Patients** |
| --- | --- |
| Stroke  Acute Myocardial Infarction (AMI) | 35% [7]  100% < 28 days, 0% > 28 days [8] |
| Angina | 80% [8] |
| Heart Failure (HF) | 100% [8] |

**5. Incidence of Disease**

Disease incidence data in the Netherlands in 2011 are reported using ICD-10 and ICPC-codes (see Table S6) [9]. Incidence for stroke and HF as reported in this database are listed in Tables S6 and S10. Incidence of Ischemic Heart Disease (IHD) is, however, only reported as one group and not separated out into AMI and angina pectoris and other chronic-IHD. Reported trends for AMI and angina incidence in 2011[9] were used instead (Tables S7 and S8).

**Table S6.** Incidence data available for cardiovascular disease in the Netherlands in bold.

| Diagnosis | ICPC-Code | ICD-10 Code | Incidence Data Available |
| --- | --- | --- | --- |
| Cerebrovascular Diseases (Stroke) |  | **I60–I69** | x |
| Ischemic Heart Disease (IHD) |  | I20–I25 |  |
| Acute Myocardial Infarction (AMI) | **K75** | I21–I22 | x |
| Other acute IHD (oa-IHD) |  | I24 |  |
| Chronic IHD |  | I20, I25 |  |
| Angina pectoris | **K74** | I20 | x |
| Other chronic IHD | **K76** | I20, I25.1–I25.9 | x |
| Other heart disease |  | I26–I51 |  |
| Heart Failure (HF) | K77 | I50 | x |

**Table S7.** Incidence of total stroke cases, stroke survivors after 28 days, with a medium- or long-term disability in 2011. ICD-10: code I60–I69 [9].

|  | **Total Stroke** | | **Stroke Survivors > 28 Day** | | **Stroke with Long-Term Disability** | | **Stroke with 6 Months Disability** | |
| --- | --- | --- | --- | --- | --- | --- | --- | --- |
|  | **Men** | **Women** | **Men** | **Women** | **Men** | **Women** | **Men** | **Women** |
| 20–29 | 76 | 57 | 68 | 51 | 24 | 18 | 44 | 33 |
| 30–39 | 237 | 184 | 213 | 165 | 74 | 58 | 138 | 107 |
| 40–49 | 811 | 619 | 727 | 555 | 255 | 194 | 473 | 361 |
| 50–59 | 1790 | 1382 | 1606 | 1240 | 562 | 434 | 1044 | 806 |
| 60–69 | 3310 | 2615 | 2969 | 2346 | 1039 | 821 | 1930 | 1525 |
| 70–79 | 3736 | 3514 | 3351 | 3152 | 1173 | 1103 | 2178 | 2049 |
| 80+ | 3060 | 4762 | 2745 | 4272 | 961 | 1495 | 1784 | 2776 |

**Table S8.** Incidence trends of Acute Myocardial Infarction in 2011. ICPC-code: K75 [9].

| **Age (Year)** | **Men** | **Women** |
| --- | --- | --- |
| 20–29 | 11 | 6 |
| 30–39 | 110 | 50 |
| 40–49 | 818 | 314 |
| 50–59 | 2851 | 1050 |
| 60–69 | 5926 | 2367 |
| 70–79 | 5653 | 3116 |
| 80+ | 3011 | 3289 |

**Table S9.** Incidence trends of angina pectoris in 2011. ICPC-code: K74 [9].

| **Age (Year)** | **Men** | **Women** |
| --- | --- | --- |
| 20–29 | 8 | 7 |
| 30–39 | 81 | 51 |
| 40–49 | 602 | 317 |
| 50–59 | 2098 | 1060 |
| 60–69 | 4360 | 2390 |
| 70–79 | 4159 | 3146 |
| 80+ | 2215 | 3321 |

**Table S10.** Incidence of Heart Failure in 2011. ICPC-code: K77 [9].

| **Age (Year)** | **Men** | **Women** |
| --- | --- | --- |
| 20–29 | 1 | 1 |
| 30–39 | 29 | 26 |
| 40–49 | 222 | 197 |
| 50–59 | 662 | 594 |
| 60–69 | 1735 | 1584 |
| 70–79 | 3481 | 3770 |
| 80+ | 5022 | 9258 |

Mortality rates due to stroke (Table S11), AMI (Table S12), angina (Table S13), and HF (Table S14), were retrieved from the same database [9].

**Table S11.** Stroke mortality. ICD-10 code: I60–I69 [9]

| **Age (Year)** | **Men** | **Women** |
| --- | --- | --- |
| 20–29 | 5 | 3 |
| 30–39 | 9 | 14 |
| 40–49 | 73 | 74 |
| 50–59 | 173 | 147 |
| 60–69 | 392 | 285 |
| 70–79 | 931 | 802 |
| 80+ | 1644 | 3440 |

**Table S12.** Acute Myocardial Infarction mortality. ICD-10 code: I21–I22 [9].

| **Age (Year)** | **Men** | **Women** |
| --- | --- | --- |
| 20–29 | 5 | 1 |
| 30–39 | 29 | 8 |
| 40–49 | 156 | 53 |
| 50–59 | 408 | 108 |
| 60–69 | 719 | 249 |
| 70–79 | 984 | 587 |
| 80+ | 1295 | 1637 |

**Table S13.** Angina mortality. ICD-10: code I20 [9].

| **Age (Year)** | **Men** | **Women** |
| --- | --- | --- |
| 20–29 | 0 | 0 |
| 30–39 | 1 | 0 |
| 40–49 | 1 | 0 |
| 50–59 | 7 | 0 |
| 60–69 | 6 | 4 |
| 70–79 | 15 | 11 |
| 80+ | 42 | 50 |

**Table S14**. Heart Failure mortality. ICD-10: code I50 [9].

| **Age (Year)** | **Men** | **Women** |
| --- | --- | --- |
| 20–29 | 1 | 1 |
| 30–39 | 6 | 2 |
| 40–49 | 29 | 11 |
| 50–59 | 67 | 28 |
| 60–69 | 167 | 82 |
| 70–79 | 541 | 373 |
| 80+ | 1551 | 2936 |

**6. Disability Weight Factors**

We used the disability weight (DW) factors most recently reported by WHO that were based on the Global Burden of Disease study estimates 2000–2011. We calculated a *weighted average* of disability for the acute phase of stroke and AMI in line with “Global and regional burden of stroke during 1990–2010: findings from the Global Burden of Disease Study 2010”. Disability weights used in the calculations are summarized in Table S15.

*Stroke*: A weighted average DW was calculated for 8 days average stay in a Dutch hospital [10]; a DW of 0.1 for the first 3 days after stroke during the hyperacute phase and a DW of 0.9 the next 5 days [11] resulting in a weighted average DW of 0.600. However, the most recently reported DWs for stroke do not (yet) give a weighted average DW for chronic stroke disability of survivors beyond one month. We therefore used the weighted average DW of 0.248 which is a composite DW for all stroke survivors from the DWs of individual severities, using frequency-weighted averaging based on the Global Burden of Disease 2004 data [12].

*AMI*: A weighted average DW was calculated for an average 6,5 days stay in a Dutch hospital [10]; 0.422 for the first 2 days after AMI and 0·056 for the next 4,5 days yielding a weighted average DW of 0.169 [8].

*Angina*: Averaging over the severity distributions, and using an angina disability distribution of mild (27% of angina patients), moderate (19%), and severe (54%), the weighted average DW for angina was 0.113 [8].

*Heart failure*: Averaging over the severity distributions, and using a heart failure disability distribution of mild (23% of heart failure), moderate (22%), and severe (55%), the weighted average DW for heart failure was 0.126 [8].

**Table S15.** Disability Weight factors used for acute and chronic morbidity.

|  | **Acute** | **6-Month** | **Chronic** |
| --- | --- | --- | --- |
| Stroke | 0.600 (weighted) [11] | 0.248 (weighted) [12] | 0.248 (weighted) [12] |
| AMI ^1^ | 0.169 (weighted) [8] |  |  |
| Angina |  |  | 0.113 (weighted) [8] |
| HF ^2^ |  |  | 0.126 (weighted) [8] |

^1^ Acute Myocardial Infarction; ^2^ Heart Failure.

**7. Duration of Disease**

The duration of the acute phase of stroke was on average 8 days, as derived from data of the Netherlands Heart Association [10]. Stroke has two phases, an acute phase that often results in hospitalization and lasts less than 28 days, and a chronic phase, which may be reversible in some patients lasting on average 6 mouths, and irreversible and life-long in other patients considered to involve permanent disabilities. AMI involves only a disability in the acute phase that was estimated to last the average hospital stay of these patients of 6.5 days [6]. Angina was assumed to cause a permanent disability. We calculated the duration of heart failure by Duration = Incidence/Prevalence data as both prevalence and incidence data have been reported for the Netherlands in 2011 [9]. Disease duration as used in the calculation are summarized in Table S16.

**Table S16**. Durations used for acute and chronic morbidity.

|  | **Acute < 28 Days** | **Intermediate** | **Chronic** |
| --- | --- | --- | --- |
| Stroke | 8 days [10] | 6 mouths | Remaining life |
| AMI ^1^ | 6.5 days [6] |  |  |
| Angina |  |  | Remaining life |
| HF ^2^ |  |  | 5.2–5.5 years [10] ^3^ |

^1^ Acute Myocardial Infarction; ^2^ Heart Failure; ^3^ Calculated from prevalence/incidence.

**8. Relationships Salt Intake and Systolic Blood Pressure**

The literature was searched for meta-analyses that reported on the relationship between salt intake and Systolic Blood Pressure (SBP). Inclusion criteria were; no more than 10 years old (≥2005), sodium intake based on 24 h urine collection, and at least 4 weeks of intervention.
We identified three meta-analyses fulfilling these criteria (Table S17). One meta-analysis was based on the results of 167 studies but did not report on the quantitative relationship between salt intake and blood pressure [13]. Another meta-analysis evaluated in a sub-study 36 randomized controlled trials (RCT) and prospective cohort lasting at least 4 weeks [14]. However, no quantitative relationship between salt intake and blood pressure was reported. The meta-analysis by He *et al*. [15] was based on the results of 34 published RCTs and was the only to exclusively include intervention studies of a duration of at least 4 weeks. Therefore, this relationship between salt intake and SBP was selected for our calculations; the authors reported a SBP reduction of 0.95 mmHg SBP per 1 g lower salt intake. Data from a recent very large cohort study [16] reported a comparable reduction of 0.82 mmHg SBP per 1 g lower salt intake. A strong linear dose–response relationship between salt intake reduction and SBP reduction was identified in a recent meta-analysis based on RCT of all types and all durations [17]. Based on 0.95 mmHg lower SBP per g salt reduction, and a linear relationship between SBP and salt intake, it can be assumed that 0.11 g/day lower salt (45 mg/day sodium) intake from soups lowers SBP by 0.11 mmHg.

**Table S17.** Relation between change in salt intake and Systolic Blood Pressure reported in different meta-analysis.

| **Reference** | **Sodium Intake Based on** | **Duration Sodium Reduction** | **Number and Type of RCT ^1^ Included** | **Number of Subjects** | **Salt Reduction (g/day)** | **Reduction SBP** ^2^ **(mmHg)** | **Reduction SBP (mmHg) per g Salt** |
| --- | --- | --- | --- | --- | --- | --- | --- |
| Graudal *et al*. Cochrane 2011 [13] | Urinary excretion | ≥4 week | #167 All types | 3.377 | Not reported |  |  |
| Aburto *et al*. BMJ 2013 [14] | Urinary excretion | ≥4 week | #36 Intervention + prospective cohort | 6.736 | <2 g/day *vs*.  ≥2 g/day | 3.47 |  |
| He *et al*. Cochrane 2013 [15] | Urinary excretion | ≥4 week | #34 Intervention | 3.230 | 4.4 | 4.18 | 0.95 |

^1^ Randomized Controlled Trial; ^2^ Systolic Blood Pressure.

**9. Relationships between Systolic Blood Pressure and Stroke, IHD, and HF**

No meta-analysis was identified that reported on age group-specific relative risks (RR) reduction of mortality from stroke, IHD, and HF related to SBP lowering in cohort studies or salt-lowering intervention studies. The RR reductions for incidence of stroke and IHD per 30 mmHg lowering of SBP have been reported in a meta-analysis of 147 RCTs of blood pressure-lowering drugs recording strokes and IHD events [18] (Tables S18 and S19). The RR reductions were reported for the age of 50–79 years per 10 year age group. The RR reductions decreased with age, being approximately log-linear with age. The RR reductions for the age 20–29 years, 30–39 years, 40–49, and 80+ years were not available and modelled assuming a log-linear relationship with age.

The RR reductions of HF incidence and HF mortality attributable to lowering SBP have not been reviewed to our knowledge. Therefore, the RR reductions were derived from different studies [19–24] (Table S20).

A log-linear relation between SBP lowering and disease incidence was assumed to predict incidence of stroke, angina, and AMI with a SBP reduction of 0.11 mmHg SBP from soups [25].
For example, to obtain the RR for a 0.11 mmHg lower usual SBP with lower sodium intake, the RR for a 30 mm Hg lower usual SBP was raised to the power 0.11/30.

**Table S18.** Relative Risk of stroke incidence per 30 mmHg Systolic Blood Pressure reduction [18], and calculated Relative Risk of stroke with 45 mg/day lower sodium assuming 0.11 mmHg Systolic Blood Pressure lowering.

| **Age (Year)** | **Population** | **RR ^1^ Stroke Incidence per  30 mmHg Lower SBP ^2^** | **RR Stroke Incidence per  0.11 mmHg Lower SBP** |
| --- | --- | --- | --- |
| 20–29 | 2,036,267 | 0.18 | 0.994 |
| 30–39 | 2,126,332 | 0.19 | 0.994 |
| 40–49 | 2,594,217 | 0.20 | 0.994 |
| 50–59 | 2,286,566 | 0.22 | 0.995 |
| 60–69 | 1,894,212 | 0.27 | 0.995 |
| 70–79 | 1,136,839 | 0.35 | 0.996 |
| 80+ | 667,547 | 0.47 | 0.997 |

^1^ Relative Risk; ^2^ Systolic Blood Pressure.

**Table S19.** Relative Risk of Acute Myocardial Infarction and angina incidence per 30 mmHg Systolic Blood Pressure reduction [18], and calculated Relative Risk of Acute Myocardial Infarction and angina with 45 mg/day lower sodium assuming 0.11 mmHg Systolic Blood Pressure lowering.

| **Age (Year)** | **Population** | **RR ^1^ AMI  ^2^/Angina Incidence  per 30 mmHg Lower SBP ^3^** | **RR AMI/Angina Incidence  per 0.11 mmHg Lower SBP** |
| --- | --- | --- | --- |
| 20–29 | 2,036,267 | 0.32 | 0.996 |
| 30–39 | 2,126,332 | 0.33 | 0.996 |
| 40–49 | 2,594,217 | 0.34 | 0.996 |
| 50–59 | 2,286,566 | 0.36 | 0.996 |
| 60–69 | 1,894.212 | 0.40 | 0.997 |
| 70–79 | 1,136,839 | 0.47 | 0.997 |
| 80+ | 667,547 | 0.55 | 0.998 |

^1^ Relative Risk; ^2^ Acute Myocardial Infarction; ^3^ Systolic Blood Pressure.

**Table S20.** Relative Risk of Heart Failure incidence per 10 mmHg Systolic Blood Pressure reduction [19–24] and calculated Relative Risk of Heart Failure with 45 mg/day lower sodium assuming 0.11 mmHg Systolic Blood Pressure lowering.

| **Age (Year)** | **Population** | **RR ^1^ HF ^2^ Incidence per  10 mmHg Lower SBP ^3^** | **RR HF Incidence per  0.11 mmHg Lower SBP** |
| --- | --- | --- | --- |
| 20–29 | 2,036,267 | 0.755 | 0.997 |
| 30–39 | 2,126,332 | 0.755 | 0.997 |
| 40–49 | 2,594,217 | 0.756 | 0.997 |
| 50–59 | 2,286,566 | 0.754 | 0.997 |
| 60–69 | 1,894,212 | 0.801 | 0.998 |
| 70–79 | 1,136,839 | 0.845 | 0.998 |
| 80+ | 667,547 | 0.933 | 0.999 |

^1^ Relative Risk; ^2^ Heart Failure; ^3^ Systolic Blood Pressure.

In one meta-analysis of 107 RCTs [17], proportional effects RR reductions in cardiovascular mortality related to SBP lowering were reported, however, not in a quantitative manner, and could therefore not be used. Another meta-analysis by Lewington *et al*. [25] included prospective observational studies in which data on blood pressure were recorded at baseline and excluding studies selecting participants with a history of stroke or heart disease. The meta-analysis reported RR reductions of stroke and IHD-related mortality per 20 mmHg lowering of SBP per age group (50–59, 60–69, 70–79 and
80–89 year) [25] (Tables S21 and S22). Relative risk reductions for HF-related mortality per g salt reduction have not been reviewed except for a meta-analyses of intervention studies [26], which has later been retracted [27]. By lack of data, the RR of HF mortality was assumed similar as for HF incidence [19–24] (Table S23).

A log-linear relation between SBP lowering and disease mortality or incidence was assumed to predict mortality of stroke, angina, and MI with a SBP reduction of 0.11 mmHg SBP from soups [25].

**Table S21.** Relative Risk of stroke mortality per 20 mmHg Systolic Blood Pressure reduction [25], and calculated Relative Risk of stroke mortality with 45 mg/day lower sodium assuming 0.11 mmHg Systolic Blood Pressure lowering.

| **Age (Year)** | **Population** | **RR ^1^ Stroke Mortality per  20 mmHg Lower SBP ^2^** | **RR Stroke Mortality  per 0.11 mmHg** |
| --- | --- | --- | --- |
| 20–29 | 2,036,267 | 0.36 | 0.994 |
| 30–39 | 2,126,332 | 0.36 | 0.994 |
| 40–49 | 2,594,217 | 0.36 | 0.994 |
| 50–59 | 2,286,566 | 0.38 | 0.995 |
| 60–69 | 1,894,212 | 0.43 | 0.995 |
| 70–79 | 1,136,839 | 0.50 | 0.996 |
| 80+ | 667,547 | 0.67 | 0.998 |

^1^ Relative Risk; ^2^ Systolic Blood Pressure.

**Table S22.** Relative Risk of Acute Myocardial Infarction/angina mortality per 20 mmHg Systolic Blood Pressure reduction [25], and calculated Relative Risk of Acute Myocardial Infarction/angina mortality with 45 mg/day lower sodium assuming 0.11 mmHg Systolic Blood Pressure lowering.

| **Age (Year)** | **Population** | **RR ^1^ AMI ^2^/Angina Mortality per 20 mmHg Lower SBP ^3^** | **RR AMI/Angina Mortality per 0.11 mmHg Lower SBP** |
| --- | --- | --- | --- |
| 20–29 | 2,036,267 | 0.48 | 0.996 |
| 30–39 | 2,126,332 | 0.49 | 0.996 |
| 40–49 | 2,594,217 | 0.49 | 0.996 |
| 50–59 | 2,286,566 | 0.50 | 0.996 |
| 60–69 | 1,894,212 | 0.54 | 0.997 |
| 70–79 | 1,136,839 | 0.60 | 0.997 |
| 80+ | 667,547 | 0.67 | 0.998 |

^1^ Relative Risk; ^2^ Acute Myocardial Infarction; ^3^ Systolic Blood Pressure.

**Table S23.** Relative Risk of Heart Failure mortality per 10 mmHg reduction (based on [19–24]) and calculated Relative Risk of Heart Failure mortality with 45 mg/day lower sodium assuming 0.11 mmHg Systolic Blood Pressure lowering.

| **Age (Year)** | **Population** | **RR ^1^ HF ^2^ Mortality per  10 mmHg Lower SBP ^3^** | **RR HF Mortality per  0.11 mmHg Lower SBP** |
| --- | --- | --- | --- |
| 20–29 | 2,036,267 | 0.76 | 0.997 |
| 30–39 | 2,126,332 | 0.76 | 0.997 |
| 40–49 | 2,594,217 | 0.76 | 0.997 |
| 50–59 | 2,286,566 | 0.75 | 0.997 |
| 60–69 | 1,894,212 | 0.80 | 0.998 |
| 70–79 | 1,136,839 | 0.84 | 0.998 |
| 80+ | 667,547 | 0.93 | 0.999 |

^1^ Relative Risk; ^2^ Heart Failure; ^3^ Systolic Blood Pressure.

**10. Model Used**

The model used to calculate the effect of sodium lowering on incidence, mortality, and DALYs related to stroke, AMI, angina, and HF, is shown in Figures S1, S2 and S3, respectively.


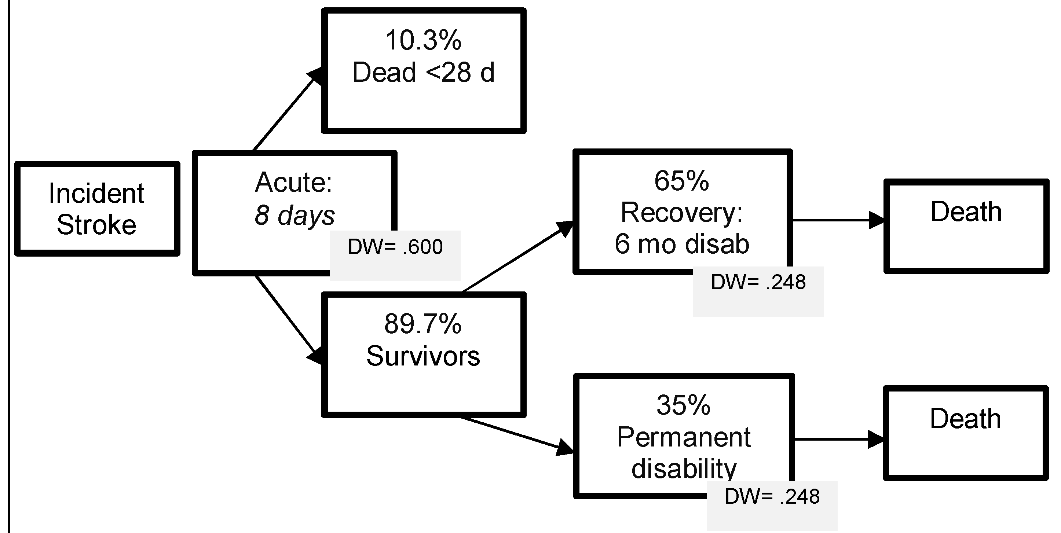


**Figure S1.** Stroke model.

**
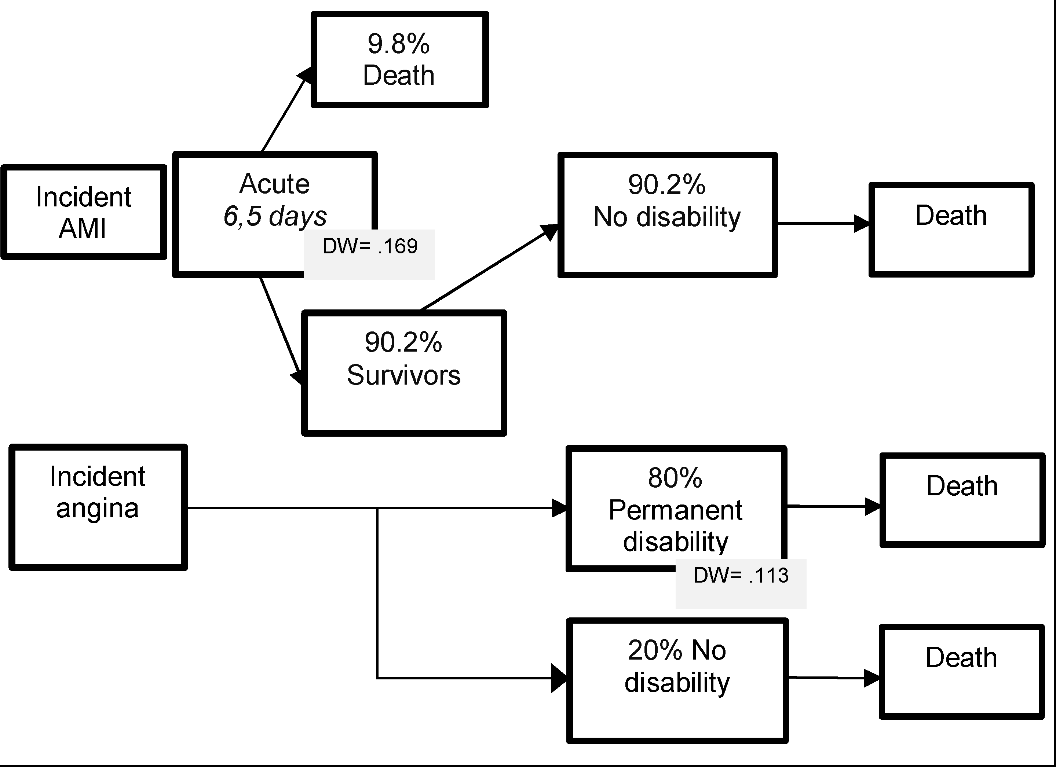
**

**Figure S2.** Acute Myocardial Infarction and angina model.

**
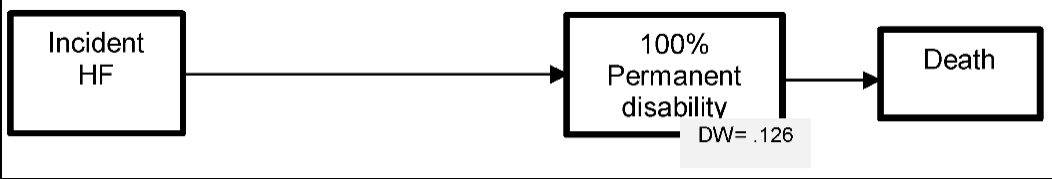
**

**Figure S3.** Heart Failure model.

11. Calculations

**Table S24.** Calculated Years of Life Lost (YLL) due to premature mortality and Years Lived with Disability (YLD), and total DALYs with usual sodium intake, with sodium reduction and change with sodium reduction in the Netherlands in 2011.

| ***Usual Sodium*** | ***Population*** | ***YLL*** | ***YLD*** | ***DALYs*** |
| --- | --- | --- | --- | --- |
| Males | 6,241,488 | 111,477 | 41,351 | 152,827 |
| Females | 6,500,492 | 117,280 | 36,780 | 154,059 |
| Total population | 12,741,980 | 228,756 | 78,130 | 306,887 |
| *Sodium reduction* | *Population* | *YLL* | *YLD* | *DALYs* |
| Males | 6,241,488 | 111,135 | 41,228 | 152,363 |
| Females | 6,500,492 | 117,023 | 36,682 | 153,704 |
| Total population | 12,741,980 | 228,158 | 77,909 | 306,067 |
| *Change* | *Population* | *YLL* | *YLD* | *DALYs* |
| Males | 6,241,488 | 341 | 257 | 598 |
| Females | 6,500,492 | 123 | 98 | 221 |
| Total | 12,741,980 | 464 | 355 | 819 |

**References**

1. Health Statistics and Health Information Systems. Available online: http://www.who.int/healthinfo/
   global_burden_disease/tools_national/en/ (accessed on 13 July 2015)
2. Prüss-Üstün, A.; Mathers, C.; Corvalán, C.; Woodward, A. Introduction and Methods: Assessing the Environmental Burden of Disease at National and Local Levels, Chapter 3. In *The Global Burden of Disease Concept*; World Health Organization (WHO): Geneva, Switzerland, 2003;
   pp. 27–40.
3. Van Rossum, C.T.M.; Fransen, H.P.; Verkaik-Kloosterman, J.; Buurma, E.M.; Ocké, M.C. *Dutch National Food Consumption Survey 2007–2010*: *Diet of Children and Adults Aged 7 to 69 Years*; National Institute for Public Health and the Environment (RIVM): Bilthoven, the Netherlands, 2011.
4. Dutch Food Composition Database. NEVO online version 2011/3.0. 2011. Available online: http://www.rivm.nl/en/Topics/Topics/D/Dutch_Food_Composition_Database (accessed on
   13 July 2015).
5. Statline. Available online: <http://statline.cbs.nl/Statweb/> (accessed on 13 July 2015).
6. *Health at a Glance 2013: OECD Indicators*; Organisation for Economic Cooperation and Development (OECD): Paris, France, 2013.
7. Lingsma, H.F.; Steyerberg, E.W.; Eijkemans, M.J.; Dippel, D.W.; Scholte Op Reimer, W.J.;
   van Houwelingen, H.C. Comparing and ranking hospitals based on outcome: Results from The netherlands stroke survey. *QJM* **2010**, *103*, 99–108.
8. Moran, A.E.; Forouzanfar, M.H.; Roth, G.A.; Mensah, G.A.; Ezzati, M.; Flaxman, A.;
   Murray, C.J.; Naghavi, M. The global burden of ischemic heart disease in 1990 and 2010: The global burden of disease 2010 study. *Circulation* **2014**, *129*, 1493–1501.
9. Feiten en Cijfers over Volksgezondheid en Zorg. Available online: https://[www.volksgez
   ondheidenzorg.info/](http://www.volksgezondheidenzorg.info/) (accessed on 13 July 2015).
10. Hart-en Vaatziekten in Nederland 2012. Cijfers over Risicofactoren, Ziekte en Sterfte, 2012. Available online: <http://webshop.hartstichting.nl/Producten/Producten.aspx> (accessed on
    15 July 2015).
11. Feigin, V.L.; Forouzanfar, M.H.; Krishnamurthi, R.; Mensah, G.A.; Connor, M.; Bennett, D.A.; Moran, A.E.; Sacco, R.L.; Anderson, L.; Truelsen, T.; *et al*. Global and regional burden of stroke during 1990–2010: Findings from the Global Burden of Disease Study 2010*. Lancet* **2014**, *383*, 245–254.
12. Hong, K.S.; Saver, J.L. Quantifying the value of stroke disability outcomes: Who global burden of disease project disability weights for each level of the modified rankin scale. *Stroke* **2009**, *40*,
    3828–3833.
13. Graudal, N.A.; Hubeck-Graudal, T.; Jurgens, G. Effects of low-sodium diet *vs.* high-sodium diet on blood pressure, renin, aldosterone, catecholamines, cholesterol, and triglyceride (Cochrane Review)*. Am. J. Hypertens*. **2012**, *25*, 1–15.
14. Aburto, N.J.; Ziolkovska, A.; Hooper, L.; Elliott, P.; Cappuccio, F.P.; Meerpohl, J.J. Effect of lower sodium intake on health: Systematic review and metaanalyses*. BMJ* **2013**, *346*, f1326.
15. He, F.J.; Li, J.; Macgregor, G.A. Effect of longer term modest salt reduction on blood pressure: Cochrane systematic review and meta-analysis of randomised trials. *BMJ* **2013**, *346*, doi:10.1136/bmj.f1325.
16. Mente, A.; O’Donnell, M.J.; Rangarajan, S.; McQueen, M.J.; Poirier, P.; Wielgosz, A.; Morrison, H.; Li, W.; Wang, X.; Di, C.; *et al.* Association of urinary sodium and potassium excretion with blood pressure. *NEJM* **2014**, *371*, 601–611.
17. Mozaffarian, D.; Fahimi, S.; Singh, G.M.; Micha, R.; Khatibzadeh, S.; Engell, R.E.; Lim, S.; Danaei, G.; Ezzati, M.; Powles, J.; *et al.* Global sodium consumption and death from cardiovascular causes. *NEJM* **2014**, *371*, 624–634.
18. Law, M.R.; Morris, J.K.; Wald, N.J. Use of blood pressure lowering drugs in the prevention of cardiovascular disease: Meta-analysis of 147 randomised trials in the context of expectations from prospective epidemiological studies. *BMJ* **2009**, *338*, b1665.
19. Vaccarino, V.; Holford, T.R.; Krumholz, H.M. Pulse pressure and risk for myocardial infarction and heart failure in the elderly. *J. Am. Coll. Cardiol.* **2000**, *36*, 130–138.
20. Chae, C.U.; Pfeffer, M.A.; Glynn, R.J.; Mitchell, G.F.; Taylor, J.O.; Hennekens, C.H. Increased pulse pressure and risk of heart failure in the elderly. *JAMA* **1999**, *281*, 634–639.
21. Haider, A.W.; Larson, M.G.; Franklin, S.S.; Levy, D. Systolic blood pressure, diastolic blood pressure, and pulse pressure as predictors of risk for congestive heart failure in the framingham heart study. *Ann. Intern. Med.* **2003**, *138*, 10–16.
22. Britton, K.A.; Gaziano, J.M.; Djousse, L. Normal systolic blood pressure and risk of heart failure in us male physicians. *Eur. J. Heart Fail.* **2009**, *11*, 1129–1134.
23. Rapsomaniki, E.; Timmis, A.; George, J.; Pujades-Rodriguez, M.; Shah, A.D.; Denaxas, S.;
    White, I.R.; Caulfield, M.J.; Deanfield, J.E.; Smeeth, L.; *et al.* Blood pressure and incidence of twelve cardiovascular diseases: Lifetime risks, healthy life-years lost, and age-specific associations in 1.25 million people. *Lancet* **2014**, *383*, 1899–1911.
24. Kalogeropoulos, A.P.; Georgiopoulou, V.V.; Agha, S.A.; Grigorios, G.; Smith, A.L.;
    Kritchevsky, S.B.; Najjar, S.; Bibbins-Domingo, K.; Sutton-Tyrrell, K.; Harris, T.B.; *et al.* Systolic blood pressure and heart failure risk in the elderly: The health, aging, and body composition study. *Circulation* **2009**, *120*, S505–S506.
25. Lewington, S.; Clarke, R.; Qizilbash, N.; Peto, R.; Collins, R.; Prospective Studies, C.
    Age-specific relevance of usual blood pressure to vascular mortality: A meta-analysis of individual data for one million adults in 61 prospective studies. *Lancet* **2002**, *360*, 1903–1913.
26. DiNicolantonio, J.J.; di Pasquale, P.; Taylor, R.S.; Hackam, D.G. Low sodium versus normal sodium diets in systolic heart failure: Systematic review and meta-analysis*. Heart* **2013**, doi:10.1136/heartjnl-2012-302337.
27. Retraction*.* Low sodium versus normal sodium diets in systolic heart failure: Systematic review and meta-analysis*. Heart*. Published Online First: 21 August 2012*. Heart* **2013**, *99*, 820, doi:10.1136/heartjnl-2012-302337ret.

© 2015 by the authors; licensee MDPI, Basel, Switzerland. This article is an open access article distributed under the terms and conditions of the Creative Commons Attribution license (http://creativecommons.org/licenses/by/4.0/).
